# Ten-year experience of tricuspid valve replacement with the St. Jude medical valve

**DOI:** 10.1038/s41598-018-35142-8

**Published:** 2018-11-09

**Authors:** Xiliang Zhu, Yi Luo, Eryong Zhang, Qi An, Xijun Xiao, Li Dong, Yingqiang Guo, Ke Dian, Zhong Wu

**Affiliations:** 1 Department of Cardiovascular Surgery, Fuwai Central China Cardiovascular Hospital, Henan Province People’s Hospital, Henan Cardiovascular Hospital and Zhengzhou University, Zhengzhou, Henan Province People’s Republic of China; 2 Department of Cardiovascular Surgery, West China Hospital, Sichuan University, Chengdu, Sichuan P. R. China

## Abstract

Bioprosthetic valves for tricuspid valve replacement (TVR) have become increasingly popular in recent years, but mechanical valves remain valuable, particularly for the patients who want to avoid reoperation for bioprostheses malfunction. The aim of this study was to review our 10-year experience in adult patients who underwent TVR with the St. Jude Medical (SJM) valve. From 2005 to 2015, 265 TVRs with SJM valves were performed at our institution. The mean age at operation was 44.1 ± 9.7 years, and 207 cases (78.1%) were female. The mean follow-up was 4.9 ± 2.7 years. Preoperative atrial fibrillation was present in 199 cases (75.1%) and ascites in 26 (9.8%). Of all cases, 88.7% were characterized as New York Heart Association class III or IV. The hospital mortality was 6.4%. There were 9 deaths (3.8%) during late follow-up. The overall survival rates were 89.2% ± 2.2% at 5 years and 86.6% ± 2.9% at 10 years. The linearized rates of valve thrombosis and bleeding events were 0.8%/patient-year and 1.5%/patient-year, respectively. Three cases (1.3%) were reoperated due to prosthetic valve thrombosis. There was no reoperation for sperivalvular leakage and structural failure. The freedom from reoperation was 98.6% ± 0.8% at 5 years and 98.6% ± 0.8% at 10 years. The SJM valve in the tricuspid position is a reliable mechanical prosthesis with a low rate of valve thrombosis and reoperation. It is a reasonable choice for the patients who require mechanical valve replacement in the tricuspid position.

## Introduction

In recent years, an increasing number of patients undergoing tricuspid valve replacement (TVR) have received bioprostheses due to the great durability in the tricuspid position and to the advantage of freeing patients from anticoagulant medication^1–3^. However, bioprostheses inevitably calcify and stenose, which may cause valve dysfunction. Therefore, the mechanical valve remains valuable in TVR, particularly for the patients who refuse to take on the risk of reoperation for bioprostheses malfunction.

The St. Jude Medical (SJM) valve (St. Jude Medical, Inc., St. Paul, MN), a low-profile bileaflet mechanical valve constructed of pyrolytic carbon, has been widely used in patients^4^. It is characterized by favorable hemodynamic properties, and a low incidence of thromboembolism^5^. As a result of excellent durability, it is preferred in young patients. Although its good clinical performances in the mitral and aortic positions have been reported^4,6^, assessment for follow-up results in the tricuspid position is limited. With this background, we reviewed our 10-year experience of 265 TVRs with SJM valves at our institution.

## Results

### Early results

Preoperative and intraoperative characteristics of the study patients are presented in Table 1. The mean age at operation was 44.1 ± 9.7 years (range, 19 to 68 years), and 207 cases (78.1%) were female. The etiology of tricuspid valve disease was as follows: rheumatic in 227 cases (85.7%), congenital in 22 (8.3%), degenerative in 7 (2.6%), endocarditis in 3 (1.1%), prosthetic dysfunction in 3 (1.1%), and traumatic in 3 (1.1%).

**Table 1:** Preoperative and Intraoperative Characteristics of the Study Patients.

Variables	Total (n = 265)	The none-mild PHT group (n = 146)	The moderate-severe PHT group (n = 119)	*p* Value
Age, years	44.1 ± 9.7	44.2 ± 9.4	44.0 ± 10.2	0.905
Female	207 (78.1%)	109 (74.7%)	98 (82.4%)	0.132
Atrial fibrillation	199 (75.1%)	104 (71.2%)	95 (79.8%)	0.107
NYHA class III or IV	235 (88.7%)	126 (86.3%)	109 (91.6%)	0.176
Presence of ascites	26 (9.8%)	9 (6.2%)	17 (14.3%)	0.027
Preoperative LAD, mm	53.5 ± 15.4	50.4 ± 15.3	56.9 ± 14.9	0.001
Preoperative LVEDD, mm	45.9 ± 9.3	45.6 ± 9.0	46.3 ± 9.7	0.560
Preoperative LVEF, %	60.5 ± 9.6	61.7 ± 9.0	59.0 ± 10.1	0.026
Etiology				<0.001
Rheumatic	227 (85.7%)	111 (76.0%)	116 (97.5%)	
Congenital	22 (8.3%)	22 (15.1%)	0	
Degenerative	7 (2.6%)	6 (4.1%)	1 (0.8%)	
Endocarditis	3 (1.1%)	1 (0.7%)	2 (1.7%)	
Prosthetic dysfunction	3 (1.1%)	3 (2.1%)	0	
Traumatic	3 (1.1%)	3 (2.1%)	0	
Previous cardiac operation	36 (13.6%)	28 (19.2%)	8 (6.7%)	<0.001
Closed mitral commissurotomy	11	3	8	
MVR	12	12	0	
MVR + AVR	6	6	0	
TVP	4	4	0	
TVR	3	3	0	
Concomitant operation	227 (85.7%)	112 (76.7%)	115 (96.6%)	<0.001
MVR	131	61	70	
MVR + AVR	93	48	45	
AVR	3	3	0	
ECC time, min	143.2 ± 40.0	135.9 ± 43.4	151.6 ± 33.7	0.001
Ventilator support time, hours	41.9 ± 76.9	36.0 ± 68.2	49.1 ± 86.2	0.179
ICU stay, hours	68.0 ± 84.0	60.0 ± 71.0	77.9 ± 97.0	0.094

AVR: aortic valve replacement; ECC: extracorporeal circulation; ICU: intensive care unit; LAD: left atrial diameter; LVEF: left ventricular ejection fraction; LVEDD: left ventricular end-diastolic dimension; MVR: mitral valve replacement; TVP: tricuspid valvuloplasty; TVR: tricuspid valve replacement.

Compared with the none-mild pulmonary hypertension (PHT) group, the moderate-severe PHT group exhibited a greater prevalence of rheumatic valve disease (*p* < 0.001), larger preoperative left atrial diameter (LAD, *p* = 0.001) and lower preoperative left ventricular ejection fraction (LVEF, *p* = 0.026). More patients in the moderate-severe PHT group received TVR concomitantly with other procedures (*p* < 0.001) and required a longer extracorporeal circulation time (ECC, *p* = 0.001). However, the proportion of patients with a history of cardiac operation in the none-mild PHT group was significantly higher than the moderate-severe PHT group (*p* < 0.001).

The early postoperative results are presented in Table 2. The hospital mortality for patients undergoing TVR was 6.4%. The causes of early deaths were cardiac failure in 10 patients, multi-organ failure as a result of pulmonary infection in 4, arrhythmia (ventricular fibrillation and ventricular tachycardia) in 2, and acute renal failure in 1. The difference of hospital mortality between the none-mild PHT group (4.1%) and the moderate-severe PHT group (9.2%) was not statistically significant (*p* = 0.090).

**Table 2:** Early Postoperative Results.

	Total (n = 265)	The none-mild PHT group (n = 146)	The moderate-severe PHT group (n = 119)	*p* Value
Death	17 (6.4%)	6 (4.1%)	11 (9.2%)	0.090
Number of patients with early complications	54 (20.4%)	24 (16.4%)	30 (25.2%)	0.078
Number of early complications	61	27	34	
Low output syndrome	22	10	12	
Pneumonia	17	8	9	
Reoperation for bleeding	10	6	4	
Permanent pacemaker insertion	6	2	4	
Acute renal failure	2	0	2	
Cerebral infarction	2	1	1	
Arrhythmia	2	0	2	

PHT: pulmonary hypertension.

There were 61 cases of early complications, including 22 low cardiac output syndrome, 17 respiratory infection, 10 re-exploration for excessive postoperative bleeding, 6 permanent pacemaker implant for atrioventricuiar block, 2 acute renal failure, 2 cerebral infarction, and 2 arrhythmia. These complications occurred in 24 TVRs (16.4%) of the none-mild PHT group and 30 (25.2%) of the moderate-severe PHT group, and the difference was not statistically significant (*p* = 0.078).

Thirty-nine cases (14.7%) experienced prolonged postoperative ventilator support for more than 72 hours. Multivariate logistic analysis showed that the age (*p* = 0.027), presence of ascites (*p* = 0.025), extended ECC time (*p* = 0.005) and low LVEF (*p* = 0.027) were the independent predictors of prolonged ventilator support (Table 3).

**Table 3:** Risk Factor Analysis for Postoperative Ventilator Support time >72 hours.

Risk Factor	Univariate *p* Value	Multivariate *p* Value	Odds Ratio (95% CI)
Age	<0.001	0.027	1.054 (1.006–1.104)
Female	0.138		
Presence of ascites	0.003	0.025	3.997 (1.190–13.427)
LAD	0.024		
Low LVEF	0.000	0.027	0.954 (0.915–0.995)
Concomitant operation	0.162		
ECC time	0.001	0.005	1.018 (1.006–1.030)

LAD: left atrial diameter; LVEF: left ventricular ejection fraction; ECC: extracorporeal circulation; CI: confidence interval.

### Ten years survival

**Figure 1:**
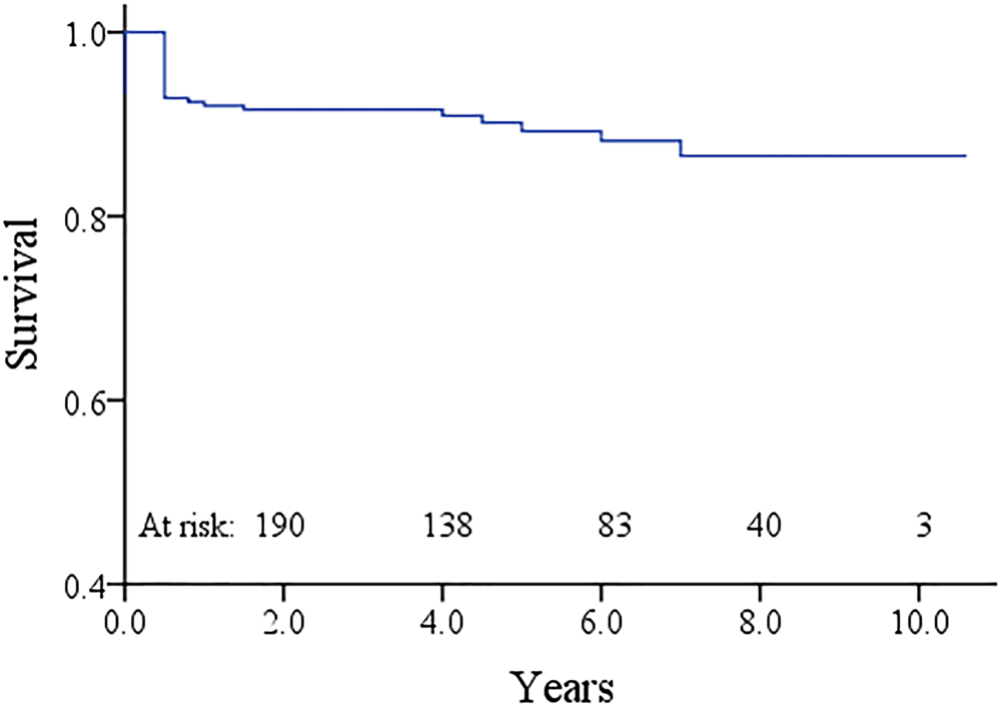
Kaplan-Meier curve estimating the overall survival of patients after TVR with SJM valves. TVR, tricuspid valve replacement; SJM, St. Jude Medical.

Of the 235 survivors, 9 deaths occurred during follow-up, yielding a late mortality rate of 3.8%. The causes of late deaths were heart failure in 3 patients, cerebral hemorrhage in 1, and prosthetic valve endocarditis in 1. However, the specific causes had not been confirmed in the remaining 4 deaths and autopsy was not conducted. For the patients with a medical history of heart failure and peripheral edema, the cause of these deaths was considered to be heart failure. Overall survival rates were 89.2% ± 2.2% at 5 years and 86.6% ± 2.9% at 10 years (Fig. 1).

**Figure 2:**
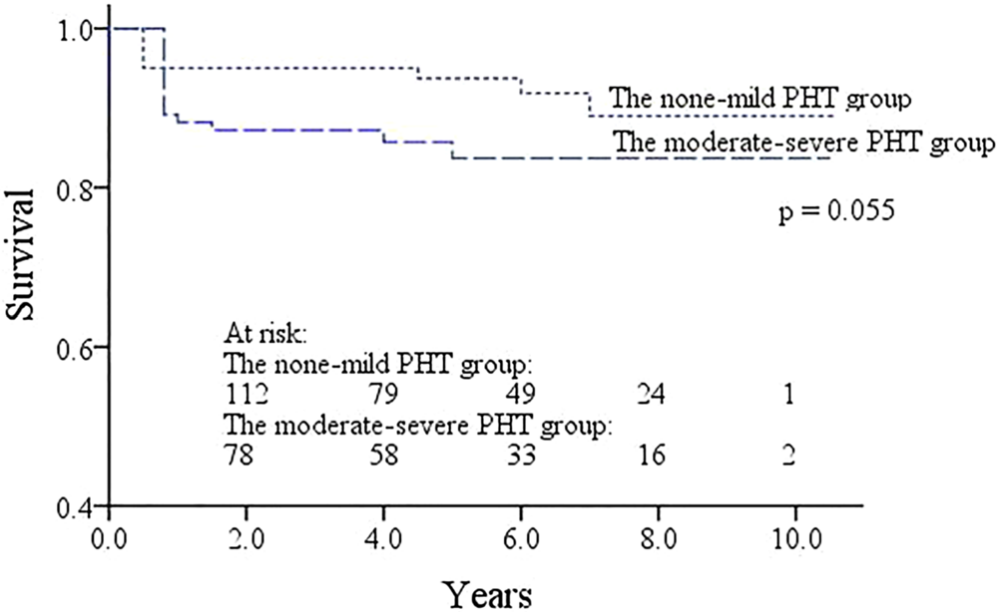
Kaplan-Meier curves estimating the late survival of patients in the none-mild PHT group and in the moderate-severe PHT group. PHT, pulmonary hypertension.

There were 4 late deaths (3.0%) in the none-mild PHT group and 5 (5.0%) in the moderate-severe PHT group. The 5 years and 10 years survival rates were 93.7 ± 2.2% and 89.0 ± 4.0% for the none-mild PHT group, and 83.7% ± 3.9% and 83.7% ± 3.9% for the moderate-severe PHT group, respectively. Although the survival rates in the none-mild PHT group were higher than the moderate-severe PHT group, the difference was not statistically significant (*p* = 0.055, Fig. 2).

### Valve thrombosis

During follow-up, valveN thrombosis occurred in 9 cases (3.8%). Of them, 7 cases were associated with an irregular use of warfarin and unreasonable prolonging the intervals of international normalized ratio (INR) monitoring (more than 3 months). The other 2 cases were confirmed to have adequate anticoagulation with a therapeutic INR.

**Figure 3:**
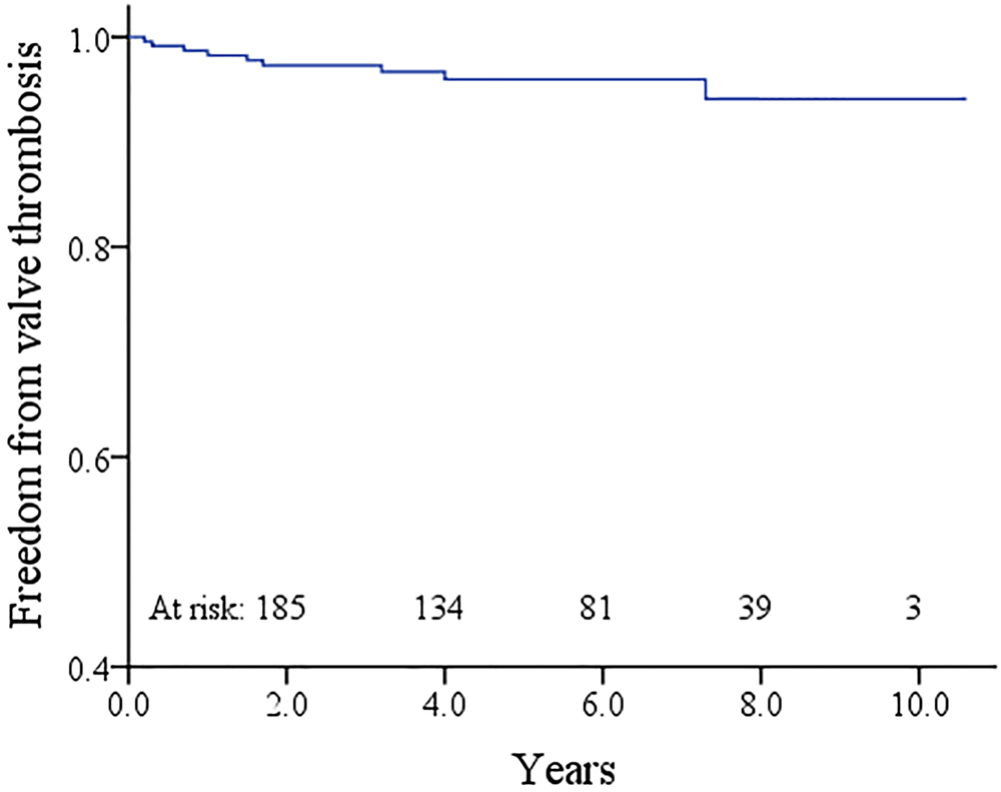
Kaplan-Meier curve estimating the freedom from valve thrombosis.

Valve replacement was successfully performed in 3 cases. As no overt symptoms related to the tricuspid valve thrombosis were observed in the remaining 6 cases, they accepted conservative management which increased the target range of INR to 2.5–3.0. Echocardiographic monitoring showed complete resolution of the thrombus within 6 months follow-up and normally functioning mechanical leaflets in 2 cases. Freedom from valve thrombosis was 96.0% ± 1.4% at 5 years and 94.1% ± 2.3% at 10 years (Fig. 3). The linearized rate of valve thrombosis was 0.8%/patient-year.

### Bleeding events

**Figure 4:**
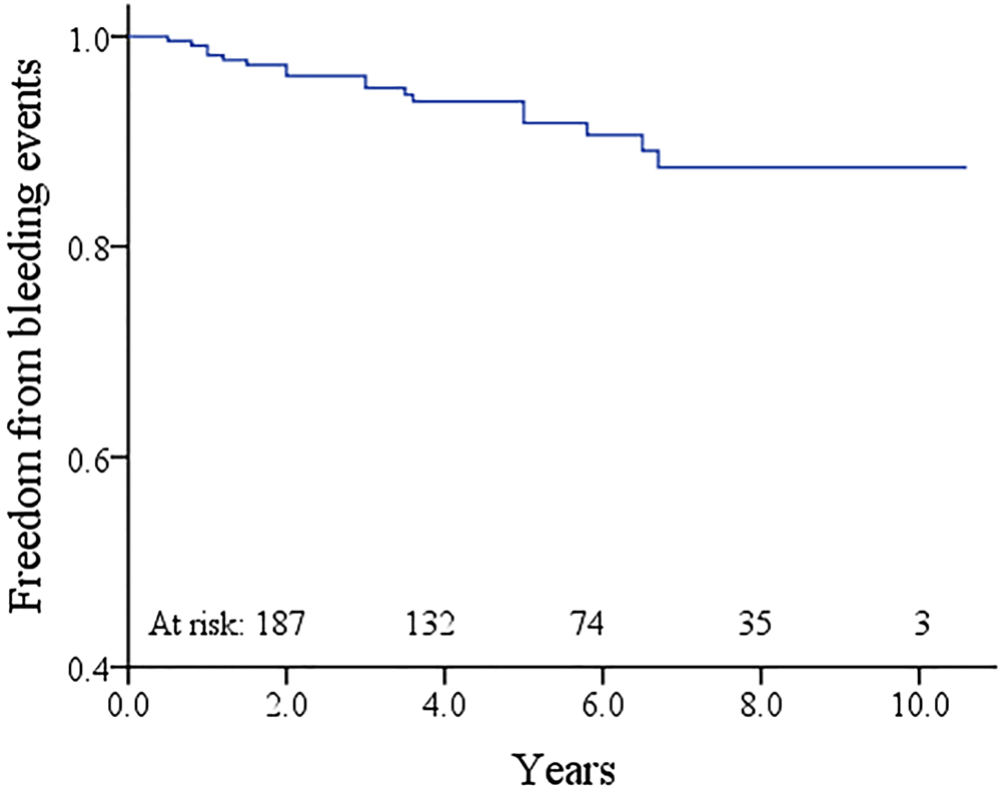
Kaplan-Meier curve estimating the freedom from bleeding events.

Bleeding events that required inpatient management or transfusion was occurred in 17 cases (7.2%). Among them, 6 cases were gastrointestinal bleeding, 3 were cerebral bleeding (1 fatal), 3 were hematuria, 2 were leg hematomas, 2 were massive epistaxis, and 1 was retinal hemorrhage. Freedom from bleeding events was 91.8% ± 2.2% at 5 years and 87.5% ± 3.2% at 10 years (Fig. 4). The linearized rate of bleeding events was 1.5%/patient-year.

### Reoperation

Reoperation was performed for 3 cases (1.3%) due to prosthetic valve thrombosis. A new SJM valve was re-implanted in 1 case, and bioprostheses were implanted in the other 2. All of them survived at the end of follow-up. The intervals to reoperation were 2 months, 4 months, and 2.8 years, respectively. Freedom from reoperation was 98.6% ± 0.8% at 5 years and 98.6% ± 0.8% at 10 years. The linearized rate of reoperation was 0.3%/patient-year. Another case of perivalvular leakage was detected after follow-up of 6.5 years, but it was unnecessary to undergo reoperation. In addition, no case of structural failure was observed.

## Discussion

TVR is uncommon in clinical practice and is reserved for few cases in which repair of the tricuspid valve is not feasible or efforts to repair it have failed^7^. A high hospital mortality rate associated with TVR was reported universally in previous studies, ranging from 10.0% to 27.6%^8–10^. The present result of 6.4% is relatively favorable compared with them. However, it is still higher than researches on mitral or aortic valve replacement^4,6^.

The reasons for such a favorable hospital mortality are varied and could be summarized as follows. First, a younger patient population is an important factor, as age was identified to be a strong predictor of hospital mortality^4,11,12^. The average age of our patients was 44.1 ± 9.7 years, while other studies were commonly more than 50 or even 60 years^9,11–14^. Second, recent advances in myocardial protection, surgical techniques and perioperative management may have had a positive effect^2,15^. Third, a low proportion of patients (13.6%) received previous open heart operations in this study^16^. Re-entry is technically challenging and is associated with a high risk of catastrophic hemorrhage and death.

Like other studies, the main cause of hospital mortality in this study was heart failure. Tricuspid valve disease is primarily asymptomatic and can be tolerated for a long period. Patients often arrive for surgery with a long history of heart disease. However, when the symptoms of right ventricular malfunction, such as peripheral edema and ascites, are manifested, the clinical condition of these patients is already into a rapidly-deteriorated stage^16,17^. We observed that the presence of ascites and low LVEF were independent predictors for more than 72 hours of ventilator support. Topilsky and colleagues^8^ reported the early results of 189 patients who had received TVR for severe tricuspid regurgitation, and they indicated that an acceptable hospital mortality can derive from a surgical correction before the onset of advanced heart failure symptoms. In recent years, our attitude toward the surgical correction of tricuspid valve disease tend to be more aggressive than ever in an aim to prevent or at least retard the progress of heart failure. Therefore, the lower hospital mortality found in this study is also related to the earlier intervention for tricuspid valve disease.

The survival rate at 10 years in this study was 86.6% ± 2.9%, which is higher than that reported in other series, ranging from 59.9% to 84.3%^10,16,18^. Consistent with previously published studies^8,11^, we found that the main cause of late death was progressive heart failure and only 2 deaths occurred as a result of valve-related complications. Some authors^5,16,19^ have indicated that patient-related characteristics, such as heart failure and the presence of ascites, were independent predictors of long-term mortality, and the type of implanted prosthesis (mechanical valve and bioprosthesis) in the tricuspid position did not affect the early and long-term results.

Garatti and colleagues^12^ reviewed 90 patients who underwent TVR with a mechanical valve or a bioprosthesis during 25 years of follow-up. Nine of 13 late deaths were associated with heart failure and only 1 patient died from cerebral hemorrhage. The late deaths were dominantly dependent from non-valve-related events. Chang and colleagues^16^ revealed a similar long-term mortality of bioprosthetic and mechanical valves, and indicated that the progression of heart failure is the most frequent cause of late death. These data demonstrate that late deaths are more attributed to patient-related characteristics than valve-related factors by the implanted prosthesis in the tricuspid position. Although the literatures have demonstrated the elevated pulmonary hypertension was an independent predictor for pathetic long-term outcomes^12,20^, not so in this study. The reason might be due to a higher previous open heart surgery rate in the none-mild PHT group than moderate-severe PHT group.

Valve thrombosis is considered the Achilles’ heel of mechanical valves in the tricuspid position. Its linearized rate ranges from 0.5% to 6.8%/patient-year according to other studies^16,18,21–24^. The present result of 0.8%/patient-year is comparable with or even lower than these reports. A higher prevalence of valve thrombosis in caged ball and tilting disc prostheses has been generally mentioned^21^.

The SJM valve was introduced as an alternative to last-generation mechanical valves with preeminent hemodynamics and low incidence of thromboembolism^4,5^. In the case of valve dysfunction for thrombosis, the bileaflet SJM valve may present hemodynamic advantages over the caged ball and tilting disc prostheses: one of the leaflets may still partially open and close to avoid catastrophic outcomes^25^. Nakano and colleagues^23^ observed that valve thrombosis occurred in only 1 out of 39 patients with the SJM valve in the tricuspid position over a 13.8-year follow-up. The result from Singh and colleagues^25^ is more promising because there was no thrombosis in 14 patients with 7 to 10 years of follow-up. However, in a retrospective study by Kawano and his colleagues^24^, 6 of 19 SJM valves after TVR were detected with valve thrombosis (2.9%/patient-years), and they emphasized that thrombosis was more likely to occur in valves of the tricuspid position. In our view, the higher thrombogenicity of the SJM valve may have been caused by the setting of a lower target INR (1.5–2.0). The irregular use of warfarin and the unreasonable prolonging of the intervals of INR monitoring were the major causes of valve thrombosis in this study. Therefore, the strict control of the anticoagulation intensity and the regular education of patients are important to avoid thrombotic complications^12,16^.

Bleeding was the main valve-related complication in this study. The present linearized rate of bleeding events was 1.5%/patient-year, which is higher than a previous series with SJM in other valve positions (0.3–1.0%/patient-year)^6,26^. However, it seems to be a satisfactory result when compared with other mechanical valves in tricuspid valve position^13,27^. In China, after mechanical aortic valve replacement (AVR) and/or mitral valve replacement (MVR), low-intensity anticoagulation with a target INR less than 2.5 (AVR: 1.3–1.8; MVR: 1.8–2.3; AVR and MVR: 1.8–2.3) is recommended for decreasing the risk of bleeding^28^. Given the higher incidence of valve thrombosis in the tricuspid position, moderate-intensity anticoagulation (INR 2.0–3.0) is recommended in our hospital for patients who received TVR with a mechanical valve. The results of this study indicated that for Chinese TVR recipients, the target INR of 2.0–3.0 was concomitant with a low incidence of valve thrombosis and an acceptable occurrence of bleeding events, but further evaluations are required to confirm these findings.

Long-term durability is one of the creditable characteristics of the SJM valves. Structural failure due to the disruption of components of the prosthetic valve was not observed in this study or in other relevant studies^23–25^. In this study, 98.6% ± 0.8% of SJM valves were free from reoperation at 10 years. All reoperations were identified for valve thrombosis, rather than structural failure and perivalvular leakage. Similar to that of other studies^23,25^, excellent durability of the SJM valves was demonstrated in this study.

Several studies have reported better durability of bioprostheses in a low-pressure chamber of the tricuspid position than in the aortic and mitral position^9,15^. However, the deficiencies for bioprostheses are late valve calcification, stenosis, and dysfunction. Nakano and colleagues^18^ detected the subclinical prosthetic dysfunction of bioprosthetic valves in 35% of patients who were followed up for more than 5 years after TVR. Chang and colleagues^16^ compared the long-term outcomes between two types of valves. Freedom from reoperation for mechanical valves at 15 years was 86.0% ± 6.2%, while that of bioprosthetic valves was only 55.1% ± 13.8%. The reoperation of TVR would bring about a high operative risk and hospital expense. As the patients in this study were characterized by a younger age and a lower income distribution, they refuse to take on the risk of reoperation for bioprostheses malfunction. Therefore, they more preferable to mechanical valves at present.

In recent years, advances in the transcatheter valve-in-valve (VIV) technology is forcing more and more surgeons to implant bioprostheses, even in young patients. It is an attractive alternatives to redo conventional open heart surgery. However, most of the available studies are mainly focus on the treatment of dysfunctional aortic bioprostheses^29^. In our opinion, more experiences in transcatheter tricuspid VIV and longer follow-up are needed before the expansion of bioprosthesis used in tricuspid position^30^. The technology of transcatheter tricuspid VIV may open a new clinical prospect in the near future for patients.

### Study limitations

There are also limitations in this study that must be realized. First, it was a retrospective observational study performed in a single institution. However, to our knowledge, it is the largest number of patients undergoing TVR with SJM valve to be studied. Second, 85.7% of patients had concomitant MVR and/or AVR, early complications such as low cardiac output syndrome, respiratory infection, reoperation for bleeding might not have been directly related to mechanical TVR, rather may be due to valve replacement in either aortic or mitral valves. Third, for an average duration of 4.9 ± 2.7 years of this study, the long-term results still need to be further followed up.

## Conclusion

This study demonstrates the excellent mid-term results of SJM valve in the tricuspid position. The incidences of valve thrombosis and reoperation were low, and deaths were more attributed to the patient-related characteristics rather than valve-related complications. Therefore, the SJM valve is a reasonable choice for the patients who require mechanical valve replacement in the tricuspid position.

## Materials and Methods

### Patients

From January 2005 to December 2015, 10202 cardiac valve replacements were performed in West China Hospital, Sichuan University. Of these operations, 5574 (54.6%) were MVR, 2666 (26.1%) were MVR and AVR, 1630 (16.0%) were AVR, and 332 (3.3%) were TVR either as an isolated procedure or combined with surgery on other valves. After patients with bioprostheses (42 cases), non-SJM mechanical valves (GK valve in 11 cases and CarboMedics valve in 9) and younger than 18 years old (5 cases) were excluded, 265 cases of TVR with SJM valves were performed in 264 adult patients (see Supplementary Fig. 1). During the same period, there were 3658 cases of tricuspid valve repair in our hospital, and the ratio of replacement to repair was 1:11.

### Surgical techniques

**Figure 5:**
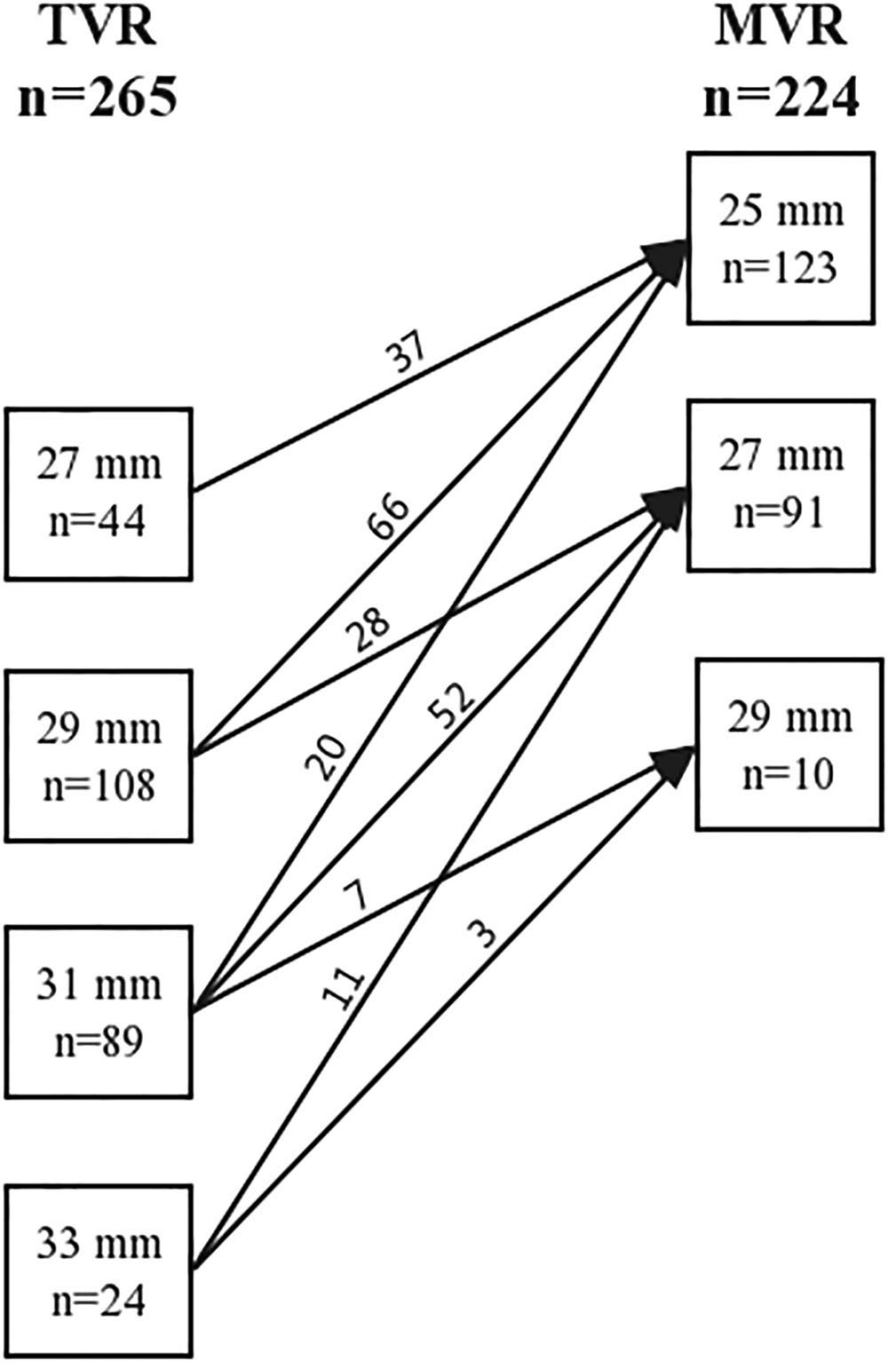
The prosthetic valve sizes of the SJM valves which were used in TVR and MVR. MVR, mitral valve replacement; TVR, tricuspid valve replacement; SJM, St. Jude Medical.

In our hospital, the first choice for patients with tricuspid valve disease is repair, and the replacement should only be considered as the last resort. A total of 261 (98.5%) TVRs were performed under cardiac arrest through median sternotomy in a conventional manner. The remaining 4 reoperative cases (1.5%) were performed while the heart was beating, and a right anterolateral thoracotomy was used to avoid retrosternal adhesions. Cardiopulmonary bypass and mild-moderate systemic hypothermia were initiated for all cases during the operations. The SJM valves were implanted with interrupted sutures. They were also used in other valve replacements, which were performed concomitantly with TVR. The prosthetic valve sizes of the SJM valves in TVR and MVR are shown in Fig. 5. Postoperative anticoagulation with warfarin was administered for all patients with the INR ranging from 2.0 to 3.0. Our anticoagulant management was monitored every month, but every 2–3 months for patients whose INR was consistently within the target range over 3 months.

### None-mild PHT vs. moderate-severe PHT groups

The preoperative PHT was calculated through Doppler echocardiography by adding the systolic pressure gradient between the right ventricle and atrium to the right atrial pressure^31^. The patients were divided into the none-mild PHT (≤50 mm Hg) group and the moderate-severe PHT (>50 mm Hg) group according to the preoperative PHT condition. Both the preoperative and intraoperative data of the two groups are shown in Table 1.

### Follow-up

Clinical follow-up was accomplished by a review of the medical records or telephone interviews with the patients themselves or their family members. The follow-up rate was 95.1% (252 of 265 cases), with an average duration of 4.9 ± 2.7 years ranging from 0 to 10.6 years. The cumulative follow-up was 1141.4 patient-years. Operative mortality was to be reported as all-cause mortality within 30 days of TVR or during the time of hospitalization^32^. The events that occurred within this time were defined as early complication. Valve-related complications associated with the prosthetic valve in the tricuspid position, including valve thrombosis, bleeding events, prosthetic valve endocarditis, and perivalvular leakage, were included in the data analysis^2,32^

### Statistical analysis

Continuous variables were described as mean ± standard deviation (SD) and were analyzed with the Student’s *t*-test. Categorical data were reported as rates and were analyzed with the Chi-squared test. Independent predictors for ventilator support time more than 72 hours were evaluated with a logistic regression model. Variables with *p* value < 0.20 in univariate analysis were incorporated into multivariate analysis. The rates of overall survival, freedom from valve-related complications and reoperations were estimated with the Kaplan-Meier method respectively, and the log-rank test was used for intergroup comparison. The incidences of valve-related complications were reported as linearized rates. A two-sided *p* < 0.05 was regarded as statistically significant. All data were analyzed with Statistical Package for Social Sciences (SPSS V17.0, Chicago, Illinois, USA).

### Ethics statement

This study followed the tenets of Declaration of Helsinki and was approved by the ethics review board of West China Hospital, Sichuan University. A waiver of consent was received from them at the same time.

## Electronic supplementary material

Supplementary Figure S1

## Data Availability

The authors declare that all the data in this manuscript are available.
