# Supplementary material for: Ten-year experience of tricuspid valve replacement with the St. Jude medical valve
Source: Sci Rep. 2018 Nov 9;8:16654. doi: 10.1038/s41598-018-35142-8 (PMC6226498; doi:10.1038/s41598-018-35142-8)
Supplement: Supplementary file 1 — Supplementary Figure S1 [file 41598_2018_35142_MOESM1_ESM.doc]

**Supplementary Information**

**Ten-year experience of tricuspid valve replacement with the St. Jude medical valve**

Xiliang Zhu^a, b＊^, Yi Luo^b＊^, Eryong Zhang^b^, Qi An^b^, Xijun Xiao^b^, Li Dong^b^, Yingqiang Guo^b^, Ke Dian^b^, and Zhong Wu^b^

^a^Department of Cardiovascular Surgery, Fuwai Central China Cardiovascular Hospital, Henan Province People's Hospital, Henan Cardiovascular Hospital and Zhengzhou University, Zhengzhou, Henan Province, People's Republic of China;

^b^Department of Cardiovascular Surgery, West China Hospital, Sichuan University, Chengdu, Sichuan, P.R. China;

^＊^Xiliang Zhu and Yi Luo contributed equally to this article.

This file includes

Supplementary Figures S1

**Supplemental Figures:**


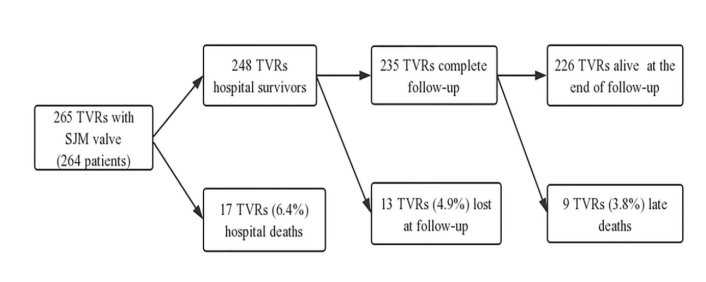


**Supplementary Figure S1. Flow diagram.** TVR, tricuspid valve replacement; SJM, St. Jude Medical.
